# Evaluation of immune responses in HIV infected patients with pleural tuberculosis by the QuantiFERON^® ^TB-Gold interferon-gamma assay

**DOI:** 10.1186/1471-2334-8-35

**Published:** 2008-03-14

**Authors:** Kamaldeen Baba, Steinar Sørnes, Anwar A Hoosen, Jacob M Lekabe, Mathew J Mpe, Nina Langeland, Anne M Dyrhol-Riise

**Affiliations:** 1 Institute of Medicine, University of Bergen, 5021 Bergen, Norway; 2 Center for International Health, University of Bergen, 5021 Bergen, Norway; 3 Department of Microbiological Pathology, University of Limpopo, Pretoria, South Africa; 4 Department of Hematology, University of Limpopo, Pretoria, South Africa; 5 Department of Intensive Care Medicine, University of Limpopo, Pretoria, South Africa; 6 Department of Medicine, Haukeland University Hospital, 5021 Bergen, Norway

## Abstract

**Background:**

Diagnosis of tuberculous (TB) pleuritis is difficult and better diagnostic tools are needed. New blood based interferon-gamma (IFN-γ) tests are promising, but sensitivity could be low in HIV positive patients. The IFN-γ tests have not yet been validated for use in pleural fluid, a compartment with higher level of immune activation than in blood.

**Methods:**

The QuantiFERON TB^®^-Gold (QFT-TB) test was analysed in blood and pleural fluid from 34 patients presenting with clinically suspected pleural TB. Clinical data, HIV status and CD4 cell counts were recorded. Adenosine deaminase activity (ADA) analysis and TB culture were performed on pleural fluid.

**Results:**

The patients were categorised as 'confirmed TB' (n = 12), 'probable TB' (n = 16) and 'non-TB' pleuritis (n = 6) based on TB culture results and clinical and biochemical criteria. The majority of the TB patients were HIV infected (82%). The QFT-TB in pleural fluid was positive in 27% and 56% of the 'confirmed TB' and 'probable TB' cases, respectively, whereas the corresponding sensitivities in blood were 58% and 83%. Indeterminate results in blood (25%) were caused by low phytohemagglutinin (PHA = positive control) IFN-γ responses, significantly lower in the TB patients as compared to the 'non-TB' cases (p = 0.02). Blood PHA responses correlated with CD4 cell count (r = 0.600, p = 0.028). In contrast, in pleural fluid indeterminate results (52%) were caused by high Nil (negative control) IFN-γ responses in both TB groups. Still, the Nil IFN-γ responses were lower than the TB antigen responses (p < 0.01), offering a conclusive test for half of the patients. We did not find any correlation between blood CD4 cell count and IFN-γ responses in pleural fluid.

**Conclusion:**

The QFT-TB test in blood could contribute to the diagnosis of TB pleuritis in the HIV positive population. Still, the number of inconclusive results is too high to recommend the commercial QFT-TB test for routine use in pleural fluid in a TB/HIV endemic resource-limited setting.

## Background

Tuberculosis (TB) is globally a major health burden and human immunodefiency virus (HIV) infection is a strong risk factor for the progression from latent infection to active TB. In South Africa 60% of the adult TB cases are HIV positive [1]. TB pleuritis occurs in about 30% of TB patients, the majority of cases in the HIV positive population [2].

The diagnosis of TB pleuritis is generally difficult and the acid fast bacilli [AFB] microscopy method rarely detects the tubercle bacilli, whereas culture is positive in about 40% of cases [3]. Histology of pleural biopsies could offer a sensitivity of up to 80% in immunocompetent patients [4], but expected to be much lower in HIV patients and specificity is generally low [5].

Adenosine deaminase activity (ADA) in pleural fluid is used as a marker of TB pleuritis and a meta-analysis conclude that although it has variable performance, it is still a useful test [6]. However, emphyema, rheumatoid pleurisy and malignancy may give false positive results [7], while immune suppression could give a false negative test [8]. Finally, a sensitivity as low as 17% in pleural fluid has been reported for the polymerase chain reaction (PCR) method [9].

HIV infection has changed the nature of the clinical presentation of pleural TB [2]. Patients co-infected with HIV and TB have stronger pleural reactions than HIV negative persons [10]. Further, T cells from the pleural cavity in patients with TB pleuritis are more activated than those from the peripheral blood and there seems to be a compartmentalisation of TB specific interferon-gamma (IFN-γ) producing cells in the lungs of patients with active TB [11]. T cell responses to TB antigens reside predominantly in the CD4+ T cell subset [12]. A decline in the number of CD4+ T cells and an expansion of activated memory CD8+ T cells characterise chronic HIV infection [13]. Thus, it is of importance to study the immune responses at the local site of infection in order to improve the understanding of the immunological mechanisms involved in containment and progression of TB in HIV infected patients.

TB proteins encoded by the RD-1 gene of *Mycobacterium tuberculosis *(*M. tuberculosis*) are used in commercially available IGRA (Interferon-gamma Release Assays) blood tests [QuantiFERON^®^-TB Gold In-tube, (QFT-TB) and T spot-TB^®^] [14-17]. They offer comparable high sensitivities and specificities in the diagnosis of TB in immunocompetent patients, but there is concern about the sensitivity in immunocompromised patients, especially when using the QFT-TB test [18-20]. Thus, IFN-γ based assays may give false negative TB diagnosis in endemic areas with high burden of HIV co-infection where reliable diagnostic tools are needed the most. Several studies have evaluated the new IFN-γ assays in blood from patients with active, including extrapulmonary TB, but few HIV patients or cases of pleural TB have been included and analyses have predominately been performed on blood specimens [16,19,21-24].

The ELISA technique used in the QFT-TB assay could easily be adapted to routine practice and a recent review by Gopi *et al *requests studies of the potential efficacy of IFN-γ assays in the diagnosis of pleural TB [25]. In this study we have quantified TB specific and unspecific immune responses in the pleural cavity of patients with HIV and TB co-infection by using the commercial available QFT-TB test. Further, we have evaluated the utility of this test as a diagnostic tool of TB pleuritis in a high TB and HIV endemic area.

## Methods

### Study participants

Patients presenting with pleural effusion and clinical symptoms of TB pleuritis admitted to the Dr. George Mukhari Hospital (DGM), Ga-Rankuwa, Pretoria, South Africa in the period 2004–2005 were recruited into the study.

The patients were categorised as; (i) 'confirmed TB' pleuritis (AFB microscopy or culture positive pleural fluid), (ii) 'probable TB' pleuritis (AFB microscopy and culture negative pleural fluid, ADA ≥30 U/L and/or strong clinical evidence consistent with active TB followed by a decision by a clinician to treat with tuberculosis chemotherapy [26]) and (iii) 'non-TB' pleuritis when diagnosed as malignancy or another non-TB condition. Patients with recent TB therapy (< 1 year) or treated with corticosteroids, immunosuppressive or antiretroviral therapy (ART) were not included. HIV testing was done routinely at the hospital in pleural effusion patients with suspected TB. The demographic and clinical data, HIV status and CD4 cell count were recorded for each patient.

Thoracocentesis and pleural biopsies (for only two patients) were obtained according to clinical practice at the hospital. Peripheral blood was obtained in parallel and before initiation of anti-tuberculosis therapy. The QFT-TB results were not available for the clinicians and did not influence the classification of patients or decisions concerning treatment.

The study was evaluated and approved by the Research, Ethics and Publications Committee at the DGM Hospital, University of Limpopo, South Africa (2003) and the Regional Committee for Ethics in Medical Research in Bergen, Norway (2003, REK Vest nr.185.02). All the patients received written and oral information of the study and all gave informed consent.

### Specimen preparation and culture

The pleural fluid and sputum specimens were processed according to standard laboratory routines for AFB staining of smears and culture for up to four weeks (BacT-alert, Organon, Teknika). The pleural fluid was also sent for cytological examination and analysis of ADA, using a commercial colorimetric assay kit (Diazyme General Atomics, CA) with a cutoff value for positive test of 30 U/L [27]. Pleural fluid mononuclear cells (PFMCs) were isolated from approximately 200 ml pleural fluid by density gradient centrifugation (Ficoll histopaque 1077, Sigma), washed and resuspended in RPMI media (1640, L-glutamine and HEPES supl., Sigma) to make a final concentration of 1 × 10^6 ^cells/ml.

### QuantiFERON^® ^TB Gold In-Tube assay

One ml of blood and 1 ml of PFMC suspension were added to each of the three 'QuantiFERON^®^-TB Gold (QFT-TB) In-tubes'; TB antigen (ESAT-6, CFP-10 and TB 7.7), positive mitogen control (phytohemagglutinin [PHA]) and negative control (Nil), as provided by the manufacturer (Cellestis Ltd., Victoria, Australia), mixed well and incubated at 37°C for 20 hours. The tubes were centrifuged and 500 μl of the supernatants were harvested and stored at -70°C. until the IFN-γ was measured in duplicates simultaneously in an ELISA reader. The IFN-γ concentrations (IU/ml) were calculated by the 'QFT-TB analysis Software'. When the value was >15 IU/ml, the maximum concentration that can be estimated from the standard curve, the value was set to 15 IU/ml. For blood, the QFT-TB test was interpreted according to instructions by the manufacturer. The test was positive if the TB antigen minus Nil value was ≥0.35 IU/ml. The Nil control had to be ≤8 IU/ml and PHA (positive control) minus Nil had to be ≥0.5 IU/ml or TB antigen minus Nil value had to be ≥0.35 IU/ml and ≥ 25% of the Nil for the subject to have a valid QFT-TB test. For pleural fluid, the QFT-TB test was interpreted as for blood, but except any upper cutoff value for Nil. The pleural fluid supernatants were tested in 1:1, 1:10 and 1:20 dilutions. The mean IU/ml from two parallel samples from the dilution that was within the range of the assay was used to calculate the final results. The values for the pleural fluid were then multiplied with the dilution factor in order to standardise the final results to the number of cells (IU/1 × 10^6^cells).

### Statistics

The statistical analysis was performed by SPSS 14 (SPSS Inc., Chicago, Il, USA). IFN-γ responses are shown as median and ranges. The results were analysed by exact Mann-Whitney U and Kruskal-Wallis tests (including post hoc Mann-Whitney tests with Bonferroni correction for a group of three post hoc comparisons) for comparison between the TB groups. IFN-γ responses within a TB group were analysed by exact paired sample Wilcoxon tests. The Spearman correlation coefficient was computed for associations between continuous variables. The relationships between IFN-γ responses and HIV and TB status were investigated by linear regression. A p-value of < 0.05 was considered to indicate a statistically significant difference.

## Results

### Clinical and laboratory characteristics

Thirty-four patients with clinically suspected pleural TB were enrolled in the study and the clinical and laboratory characteristics are shown in Table 1. Chest pain, night sweat and loss of weight were the most common clinical symptoms seen in 85–100% of the patients.

**Table 1:** Clinical characteristics of patients enrolled (n = 34)

**Characteristics**	**Number**
Gender	
Females	12
Males	22
Age: median (range)	39 (20–70)
Clinical symptoms: n (%)	
Chest pain	34 (100%)
Productive cough	10 (29%)
Fever	16 (47%)
Shortness of breath	16 (47%)
Night sweat	29 (85%)
Loss of weight	31 (91%)
Lymphadenopathy	27 (79%)
Oral thrush	25 (74%)
Chest x-ray infiltrates: n (%)	2 (6%)
Culture positive TB: n (%)	12 (35%)
Total TB cases: n (%)	28 (82%)
Non-TB cases: n (%)	6 (18%)
HIV positive patients: n (%)	25 (74%)
CD4 cell count HIV positive: median (range)^a)^	80 (7–328)
CD4 cell count > 200 cells/microL	2 (8%)
CD4 cell count 100–200 cells/microL	7 (28%)
CD4 cell count < 100 cells/microL	13 (52%)
CD4 cell count HIV negative: median (range)	457 (241–927)

^a) ^CD4 cell count was not available for three of the patients.

Twelve (35%) of the patients had culture 'confirmed TB' pleuritis, whereas 16 patients (47%) were diagnosed as 'probable TB' pleuritis based on ADA ≥30 U/L and/or clinical symptoms (Table 1). Tuberculosis chemotherapy was started in patients with clinical evidence consistent with active TB, but response to therapy could not be recorded since the patients were referred to outlying hospitals and lost to follow-up. Of the remaining six patients classified as 'non-TB' pleuritis, five were diagnosed with malignancy by cytology and/or histology, whereas one patient did not have a final diagnosis.

Twenty-three of the 28 TB patients (82%) were HIV positive. The median CD4 cell count was 77 cells/microL (range 7–280) and 162 cells/microL (range 7–548) for the 'confirmed TB' and 'probable TB' patients, respectively (p > 0.05). The 'non-TB' group (two HIV positive and four HIV negative) had a median CD4 cell count of 302 cells/microL (100–927), higher than the 'confirmed TB' group (p = 0.01), but not statistically different from the 'probable TB' group (p > 0.05). However, when adjusted for HIV status there was no significant difference in CD4 cell count between any of the groups.

All 'confirmed TB' patients had ADA values above 30 U/L, whereas this was the case for 15/16 of the 'probable TB' patients. The ADA concentrations were comparable in the 'confirmed TB' and 'probable TB' groups [68 U/L (range 38–133) and 60 U/L (range 29–200), respectively] but as expected significantly higher than in the 'non-TB' group [14 U/L (range 5–19), (p = 0.001)]. The ADA levels in pleural fluid were similar in the HIV positive and HIV negative patients, and we found no significant correlation between ADA values and CD4 cell counts (data not shown).

### QuantiFERON^® ^TB Gold In-Tube results

The absolute concentrations of IFN-γ in blood and pleural fluid for the 'confirmed TB', 'probable TB' and 'non-TB' cases are as given in Table 2. There were significant differences between the three study groups in TB IFN-γ responses both in blood (p < 0.001) and pleural fluid (p = 0.022) as well as in PHA (positive control) IFN-γ responses in blood (p = 0.046) and Nil IFN-γ responses (p = 0.024) in pleural fluid. In blood, the strongest TB IFN-γ responses were found in the 'probable TB' group, but both TB groups had higher values above the cutoff of the QFT-TB assay compared to the 'non-TB' patients, (p < 0.01). Nil responses in blood were low and comparable in all groups, well below the upper cutoff for valid test of 8 IU/ml. The PHA IFN-γ responses were generally low in both TB groups as compared to the 'non-TB' cases and for the 'probable TB' group the difference was statistically significant (p = 0.02). For the whole group of patients PHA IFN-γ responses in blood correlated with blood CD4 cell count (r = 0.600, p = 0.028).

**Table 2:** Interferon-gamma (IFN-γ) responses (TB antigen, PHA and nil) in blood (n = 29) and pleural fluid (n = 32)^a)^

	**TB antigens**	**Positive control (PHA)**	**Negative control (nil)**
**Blood (IU/ml)**			
Confirmed TB pleuritis (n = 12)	1.20 (0.11–11.80)^be^	1.24 (0.11–15.00)	0.22 (0.05–2.06)
Probable TB pleuritis (n = 12)	3.97 (0.32–11.05)^be^	0.50 (0.10–4.00)^c^	0.22 (0.07–1.62)
Non-TB pleuritis (n = 5)	0.13 (0.08–0.31)	15.00 (1.23–15.00)	0.10 (0.07–0.18)
			
**Pleural fluid (IU/1 × 10**^6 ^**PFMCs)**			
Confirmed TB pleuritis (n = 16)	14.40 (2.15–169.00)	11.45 (1.85–81.20)	8.90 (2.10–55.60)^d^
Probable TB pleuritis (n = 11)	19.18 (1.31–143.90)^be^	16.60 (0.95–118.00)	15.23 (0.87–103.00)^b^
Non-TB pleuritis (n = 5)	1.10 (0.13–7.30)	4.60 (2.20–9.00)	0.70 (0.14–8.80)

Data are presented as medians. The ranges are listed in brackets. PFMC = pleural fluid mononuclear cells.

^a) ^It was not possible to obtain blood from five patients and pleural fluid from two patients.

^b) ^*p *< 0.01, ^c) ^*p *= 0.02, ^d)^*p *= 0.03 compared with the 'non-TB group'. ^e) ^*p *< 0.01 compared with negative control.

The IFN-γ responses in pleural fluid are given as IU per 1 × 10^6 ^mononuclear cells in order to standardise the results. The TB specific IFN-γ responses were stronger both for the 'confirmed TB' (p = 0.05) and for the 'probable TB' (p = 0.008) groups as compared to the 'non-TB' group in this compartment. Also the concentrations of IFN-γ in the negative control samples were significantly higher in both TB groups than that found in the 'non-TB group' (p <0.05). Still, the Nil values were lower than those measured in the TB antigen samples, and for the 'probable TB' group this was statistically significant (p = 0.003). High IFN-γ Nil levels in the 'non-TB' group, were only found in the two HIV positive patients (4.2 and 8.8 IU/1 × 10^6 ^PFMCs) and not in the HIV negative malignancy patients (0.14–0.65 IU/1 × 10^6 ^PFMCs). The various IFN-γ responses in pleural fluid did not correlate to either ADA or CD4 cell count.

By using the definition of cutoff values and valid tests as described, 16 (50%) of the QFT-TB results were conclusive in pleural fluid compared to 23 (79%) in blood. This percentage varied according to HIV status (Table 3) and whether TB was confirmed by culture (Table 4). In the 'confirmed TB' group 8/12 (67%) cases were conclusive in blood and 3/11 (27%) cases were conclusive in pleural fluid. The corresponding numbers for the 'probable TB' group were 10/12 (83%) and 10/16 (63%). There was agreement between the two compartments in 15 of the cases. Seven patients showed positive QFT-TB test in blood, whereas the results were indeterminate in pleural fluid. In contrast, in two HIV positive 'confirmed TB' patients the QFT-TB was positive in pleural fluid, whereas the results in blood were indeterminate and negative, respectively. The indeterminate results in blood were all due to low PHA responses, whereas indeterminate results in pleural fluid were caused by high negative control samples. The number of cases with indeterminate QFT-TB results increased in blood as the CD4 cell count decreased below 100 cells/microL (Table 3). In contrast, there seemed to be more conclusive results in pleural fluid with low CD4 cell count.

**Table 3:** Conclusive results of the QuantiFERON^®^-TB Gold assay in peripheral blood (n = 29) and pleural fluid (n = 32) stratified by HIV status and CD4 cell count.

	HIV positive				
		
	Total	CD4 > 200	CD4 100–200	CD4 < 100	HIV negative
**Peripheral blood**	15/21 (71%)	2/2 (100%)	5/6 (83%)	7/11 (64%)	8/8 (100%)
**Pleural fluid**	10/24 (48%)	0/2 (0%)	2/6 (33%)	7/13 (54%)	6/8 (75%)

The nominator varies according to the number of patients tested by the assay in each group.

**Table 4:** Results of the QuantiFERON^®^-TB Gold assay in peripheral blood (n = 29) and pleural fluid (n = 32)

	**Total TB**	**Confirmed TB**	**Probable TB**	**Non-TB**
QFT-TB positive test				
Peripheral blood	17/24 (71%)	7/12 (58%)	10/12 (83%)	0/5 (0%)
Pleural fluid	12/27 (44%)	3/11 (27%)	9/16 (56%)	0/5 (0%)
				
QFT-TB negative test				
Peripheral blood	1/24 (4.2%)	1/12 (8.3%)	0/12 (0%)	5/5 (100%)
Pleural fluid	1/27 (3.7%)	0/11 (0%)	1/16 (6.3%)	3/5 (60%)
				
QFT-TB indeterminate test				
Peripheral blood	6/24 (25%)	4/12 (33%)	2/12 (17%)	0/5 (0%)
Pleural fluid	14/27 (52%)	8/11 (71%)	6/16 (38%)	2/5 (40%)

The nominator varies according to the number of patients tested by the assay in each group. It was not possible to obtain blood from five patients and pleural fluid from two patients.

In pleural fluid 12 (44%) of the 27 TB cases tested by the QFT-TB assay were positive, 14 (52%) gave indeterminate results whereas one was negative (3.7%) (Table 4). In blood 17 (71%) of the 24 tested TB cases were positive, six indeterminate (25%) and one negative (4.2%). Two of the 'non-TB' cases, both HIV positive, had indeterminate QFT-TB tests in pleural fluid, whereas all had conclusive negative results in blood. This gave sensitivities of the QFT-TB test in pleural fluid of 27% and 56% in the 'confirmed TB' cases and 'probable TB' cases respectively, whereas the corresponding sensitivities in blood were 58% and 83%. However, if the indeterminate results were excluded from the analyses, the sensitivities of QFT-TB were 94% and 87% in blood and 100% and 90% in pleural fluid in the 'confirmed TB' and 'probable TB' cases, respectively.

## Discussion

Tuberculous pleuritis is an AIDS defining illness and a common opportunistic infection in endemic areas like South Africa. Kaposi sarcoma and bacterial infections could also cause pleural effusion in HIV infected patients, making diagnosis difficult [28]. The majority of the TB patients in this study had low CD4 cell counts, reflecting the serious implication of this disease in sub-Saharan Africa. Further, we confirm that low CD4 cell count could also be found in HIV negative TB pleuritis patients [29].

This is to our knowledge the first study evaluating the 2^nd ^generation QFT-TB assay in the diagnosis of pleural TB, testing in parallel blood and pleural fluid from HIV infected patients. IFN-γ based assays have been studied in the diagnosis of extrapulmonary TB [16,19,21], but few studies have included HIV positive patients [18,20,30]. In patients with pleural TB, the tests have predominantly been performed on blood samples and not evaluated for directly use on pleural fluid specimens [22,23]. Finally, there has been inadequate evidence on the validity of IFN-γ assays in immunocompromised individuals, especially in countries where TB is endemic [31].

In this group of South African TB pleuritis patients with predominantly advanced HIV disease the overall sensitivity of the QFT-TB test in blood was 71% compared to only 44% in pleural fluid. The sensitivity in blood is comparable to that found in other studies of extrapulmonary TB in HIV negative patients, reporting sensitivities of IGRA-tests between 70–90% [16,19,21]. In a study of South African children with TB, the sensitivity of the ELISPOT assay was reduced from 83% in HIV negative to 73% in HIV positive children [20]. Our QFT-TB results are slightly superior to that reported by Sohn *et al *using the 1^st ^generation QFT-TB test based on PPD in 28 suspected HIV negative TB pleuritis patients [22]. Assuming lower sensitivities if immunocompromised patients were included in their study, our data suggests that the 2^nd ^generation QFT-TB is more sensitive not only in blood [17], but also in pleural fluid than the original QFT-TB test. In comparison, only 40% of our patients were confirmed TB by culture, which is comparable to other studies of HIV negative TB pleuritis patients [3]. Although the 'non-TB' control group was too small to give a reliable estimate of specificity, no false positive QFT-TB results were found in any of the compartments indicating that the QFT-TB seems to be a reasonable good 'rule-in' test.

In blood we found overall 25% indeterminate results in the TB patients due to weak mitogen PHA IFN-γ responses. The percentage of indeterminate results in blood increased as the CD4 cell count decreased, most likely caused by T cell anergy, typically seen in advanced HIV infection [32]. However, several patients had CD4 cell counts in the range of 50–100 cells/μL, but still positive QFT-TB tests both in blood and pleural fluid. In contrast, non-conclusive results could also be obtained in blood from HIV patients with rather high CD4 cell count, indicating that also the quality of the individual's immunity is a prerequisite for a conclusive test. Studies of patients on immunosuppressive therapy [19] and with HIV infection [18] have also reported high numbers of indeterminate results. These data point to the fact that inconclusive results from blood are more common in immunocompromised patients.

For the patients with 'confirmed TB' the sensitivity of QFT-TB was only 58% in blood and 27% in pleural fluid, whereas corresponding sensitivities were 83% and 56% for the culture negative 'probable TB' patients. It is reported that positive culture are more common in advanced HIV patients compared to HIV negative TB pleuritis patients [33], possibly explained by spread of *M. tuberculosis *from the lungs into the pleural space increasing the mycobacteria concentration. Since we found a correlation between CD4 cell count and PHA responses in blood, the trend towards lower CD4 cell counts in the 'confirmed TB' group could explain the larger number of indeterminate results due to low PHA responses in this group.

In contrast, in pleural fluid we found no correlation between blood CD4 cell count and PHA IFN-γ responses, and in this compartment TB and PHA responses were generally strong. Rather, indeterminate results were due to high levels of IFN-γ measured in the negative control samples. Patients with pleural TB co-infected with HIV have a higher rate of pleural inflammation than that observed in HIV negative persons [10], which could explain the high IFN-γ responses in the negative control samples. Background responses were not correlated to blood CD4 cell count, but were especially seen in HIV patients with CD4 cell counts at the upper range for this cohort. Even the two HIV patients in the 'non-TB' group had high IFN-γ concentrations in the negative control samples, yet at a lower level than seen in the TB groups. This supports previous studies reporting high levels of chronic immune activation and compartmentalisation of immune cells in lymphoid tissue and lungs of HIV patients from early disease [34,35]. A marked increase in activated memory T cells has also been found in HIV negative patients with tuberculous pleurisy [36]. In contrast, the HIV negative 'non-TB' patients with malignancy had all very low IFN-γ background levels, as earlier reported [37].

A recent meta-analysis concludes that IFN-γ is a sensitive and specific test for the diagnosis of tuberculous pleurisy [38]. However, in HIV endemic areas one should be aware of the problem with high background signals in HIV patients causing difficulties interpreting the results. Further, there seems to be a compartmentalisation of TB specific and unspecific immune responses in pleural fluid and blood, especially in HIV positive patients, as reported in our study. Thus, the cutoff value for a positive QFT-TB test could be different in pleural fluid where immune responses seem to be at a higher 'set-point' than in blood. We also observed that there was a large variation in concentration of cells as well as strength of responses between the various patients.

In the TB groups the TB antigen IFN-γ responses were significantly higher than the IFN-γ responses in the respective negative control samples, indicating that the actual TB antigen value was above background signals when a conclusive result was given. For two of the patients with a positive test in pleural fluid, QFT-TB in blood was not conclusive. Advanced HIV patients suffer from various opportunistic pulmonary infections making precise diagnosis difficult. Thus, testing QFN-TB in pleural fluid could at certain occasions help diagnosing culture negative TB pleuritis in the HIV positive population and contribute to decision making in the clinical setting. Still, further studies are needed to determine optimal culture conditions and appropriate cutoff values as well as the usefulness and cost benefit of the QFT-TB test before it could be recommended for use as a diagnostic test of TB pleuritis in a TB/HIV endemic resource-limited setting.

Our study indicates that testing blood from HIV positive patients with pleural effusion with QFT-TB could be useful and improve management of patients as culture results take time to obtain and pleural fluid sampling not always possible to perform. We have shown that despite the risk of indeterminate results in HIV patients with low CD4 cell counts, testing of blood offers a better sensitivity than in pleural fluid with a high positive predictive value. In support of this, Connell *et a*l. have reported two perinatal TB cases where culture was positive only after six weeks, but QFT-TB results were available within 48 hours resulting in adequate and successful treatment for the patients [39]. Further, in our study five TB patients had ADA in the range of 30–45 U/L. Some clinicians use ADA cutoff values at 45 U/L to exclude false positive tests [7]. Our data demonstrate that a positive OFT-TB could contribute to the TB diagnosis in patients with negative culture and ADA values close to cutoff values.

## Conclusion

The commercial QFT-TB test in blood may prove to be useful in the diagnosis of TB pleuritis in the HIV positive population in order to urge initiation of TB therapy. Although the QFT-TB test in pleural fluid in certain cases could contribute to the TB diagnosis in HIV patients with low CD4 cell counts, our data do not support the routine use of this test in pleural fluid at this stage. Thus, further and larger studies are needed to determine appropriate cutoff values as well as the usefulness and cost benefit of this test before it could be recommended for use as a diagnostic tool in a TB/HIV endemic resource-limited setting.

## Competing interests

The author(s) declare that they have no competing interests.

## Authors' contributions

KB has participated in initiation and design of the study, experimental work, analysis and interpretation of data and drafting of the manuscript. SS has participated in experimental work and revising of the manuscript. AAH has participated in initiation and design of the study, interpretation of data and revising of the manuscript. JML has participated in experimental work and revising of the manuscript. MJM has participated in initiation of the study, collection of samples and revising of the manuscript. NL has participated in initiation and design of the study, interpretation of data and revising of the manuscript. AMDR has participated in initiation and design of the study, analysis and interpretation of data and drafting of the manuscript. All authors read and approved the final manuscript.

## Pre-publication history

The pre-publication history for this paper can be accessed here:


